# Nurr1 Promotes Lung Cancer Apoptosis Via Enhancing Mitochondrial Stress and p53-Drp1 Pathway

**DOI:** 10.1515/biol-2019-0030

**Published:** 2019-07-10

**Authors:** Shu Zhao, Peng Li, Peng Wang, Jing Yang, Peng Song, Dong Zhang, Gang Zhou

**Affiliations:** 1 Department of Oncology, the Second Medical Center, Chinese PLA (People’s Liberation Army)General Hospital, Beijing, 100853,China

**Keywords:** Nurr1, A549 lung cancer, mitochondrial stress, p53, and Drp1

## Abstract

**Objective:**

Mitochondrial homeostasis is vital for the progression of lung cancer. Nurr1 has been identified as a novel mediator of mitochondrial homeostasis in several types of cancers. The aim of our study was to investigate whether Nurr1 modulates the viability of A549 lung cancer cells by inducing mitochondrial dysfunction, with a focus on the p53-Drp1 signaling pathway.

**Methods:**

western blotting, ELISA and immunofluorescence assay was used to verify the alterations of cell death. siRNA was used to determine the role of p53-Drp1 pathway in lung cancer death.

**Results:**

Nurr1 was downregulated in A549 lung cancer cells compared to normal pulmonary epithelial cells. Interestingly, overexpression of Nurr1 reduced the viability of A549 lung cancer cells by activating apoptosis and mitochondrial stress. At the molecular level, we provide data to support the regulatory effects of Nurr1 on the p53-Drp1 signaling pathway. Blockade of the p53-Drp1 signaling pathway abolished the proapoptotic action of Nurr1 on A549 cells and sustained mitochondrial homeostasis.

**Conclusion:**

Taken together, our results depict the tumor-suppressive role played by Nurr1 in A549 lung cancer in vitro and show that the anticancer effects of Nurr1 are executed via triggering of mitochondrial dysfunction and activation of the p53-Drp1 signaling pathway.

## 1 Introduction

Lung cancer is one of the leading causes of cancer-related death worldwide, accounting for 18% of all cancer-induced deaths [1, 2]. Several risk factors have been identified for the development of lung cancer, such as cigarette smoking, radiation exposure, and air pollution. Although medical advances have been made for the diagnosis and treatment of lung cancer, the mechanisms of the pathogenesis of lung cancer are far from clear [3, 4, 5].

Nurr1, a member of the orphan receptor family, has been found to be associated with a variety of cancer biological processes, including differentiation, metastasis, invasion, proliferation and apoptosis. A previous study demonstrated a strong correlation between Nurr1 expression and cancer invasion in prostatic tissues and cell lines. In addition, ultraviolet B radiation-induced skin cancer is also related to Nurr1 downregulation. From a clinical perspective, increased Nurr1 expression has been noted in gastric cancer patients, and reduced Nurr1 expression is associated with decreases in overall survival. At the molecular level, Nurr1 activation can increase the transcription and expression of tumor-suppressive genes. Accordingly, several reports have proposed that activation of Nurr1 is of the utmost importance in the design of anticancer therapies. However, the role of Nurr1 in lung cancer has not been identified. Therefore, our study aimed to verify the anticancer effects of Nurr1 overexpression in A549 lung cancer cells in vitro.

Mitochondrial function is vital for cancer cell metabolism, survival and metastasis. A well-organized mitochondrial network provides sufficient ATP to ensure cancer growth [6]. However, damaged mitochondria trigger apoptosis and can obligate cancer cells to undergo cell death [7, 8]. Accordingly, mitochondria are the target of most chemotherapeutic drugs. In addition, radiotherapy also disturbs mitochondrial structure and function in a manner dependent on the JNK signaling pathway [9]. Interestingly, the viability of A549 lung cancer cells is closely modulated by mitochondria. Mitochondrial oxidative injury, mitochondrial fission activation and mitochondrial caspase-9 upregulation work together to initiate the apoptotic program in A549 cells and ultimately cause cancer cell death. Notably, recent studies have identified Nurr1 as the upstream mediator of mitochondrial dysfunction in Parkinson’s disease, glioblastoma multiform brain tumor, and cerebral ischemia/reperfusion injury [10, 11, 12, 13, 14]. At the molecular level, Nurr1 activates mitochondrial fission, inhibits the antiapoptotic action of Bcl-2, and evokes ROS overproduction [15, 16, 17, 18]. Based on this information, we wanted to determine whether Nurr1 affected the viability of A549 lung cancer cells by modulating mitochondrial homeostasis.

Mechanistically, p53 controls cancer cell cycle transitions and mitochondrial function. Increased p53 has been found to be associated with tumor suppression in breast cancer [19], lung cancer [20], colorectal cancer [21], and gallbladder cancer [22]. In addition, p53 seems to be the downstream effector of Nurr1 [23]. Activation of the p53 pathway upregulates the expression of Drp1, and the latter induces mitochondrial stress and cell death in different disease models, such as models of endometriosis [24], ovarian cancer [25], fatty liver disease [26], and gastric cancer [27]. In the present study, we investigated whether Nurr1 regulated mitochondrial function via Drp1 in a manner dependent on the p53 signaling pathway in A549 lung cancer cells.

## 2 Methods

### 2.1 Cell culture and treatments

In the present study, the BEAS-2B cell line (American Type Culture Collection (ATCC)® no. CRL-9609™), a normal pulmonary epithelial cell line, and the A549 cell line (ATCC® no. CCL-185EMT™), a human lung cancer cell line, were used to determine the influence of Nurr1 on cancer cell viability [28]. The cells were cultured in RPMI-1640 media (Invitrogen, Carlsbad, CA, USA) with 10 % (v/v) fetal bovine serum (HyClone, Logan, UT, USA) at 37°C in a 5 % CO_2_ environment. To evaluate the influence of the p53 signaling pathway on A549 cell viability, the pathway blocker pifithrin-α (PFTα) (10 μM, Selleck, Catalog No. S2929) was used for 2 hours at room temperature to prevent p53 activation [29].

### 2.2 Western blotting

The primary antibodies used in the present study were as follows [30]: Drp1 (1:1000, Abcam, #ab56788), Fis1 (1:1000, Abcam, #ab71498), Opa1 (1:1000, Abcam, #ab42364), Mfn1 (1:1000, Abcam, #ab57602), Mfn2 (1:1000, Abcam, #ab56889), Mff (1:1000, Cell Signaling Technology, #86668), Bcl2 (1:1000, Cell Signaling Technology, #3498), Bax (1:1000, Cell Signaling Technology, #2772), caspase9 (1:1000, Cell Signaling Technology, #9504), survivin (1:1000, Cell Signaling Technology, #2808), p53 (1:1000, Cell Signaling Technology, #9282), complex III subunit core (CIII-core2, 1:1000, Invitrogen, #459220), complex II (CII-30, 1:1000, Abcam, #ab110410), complex IV subunit II (CIV-II, 1:1000, Abcam, #ab110268), Tom20 (1:1,000, Abcam, #ab186735) [31].

### 2.3 Immunofluorescence microscopy

Cells were washed twice with PBS, permeabilized in 0.1% Triton X-100 overnight at 4°C. After the fixation procedure, the sections were cryoprotected in a PBS solution supplemented with 0.9 mol/l of sucrose overnight at 4°C [32]. The primary antibodies used in the present study were as follows: Tom20 (1:1,000, Abcam, #ab186735), p53 (1:1000, Cell Signaling Technology, #9282), Drp1 (1:1000, Abcam, #ab56788), cyt-c (1:1,000; Abcam; #ab90529).

### 2.4 TUNEL staining and MTT assay

Apoptotic cells were detected with an In Situ Cell Death Detection Kit (Thermo Fisher Scientific Inc., Waltham, MA, USA; Catalog No. C1024) according to the manufacturer’s protocol [33]. Briefly, cells were fixed with 4% paraformaldehyde at 37°C for 15 min. Blocking buffer (3% H_2_O_2_ in CH_3_OH) was added to the wells, and then cells were permeabilized with 0.1% Triton X-100 in 0.1% sodium citrate for 2 min on ice. The cells were incubated with TUNEL reaction mixture for 1 h at 37°C. DAPI (Sigma-Aldrich, St. Louis, MO, USA) was used to counterstain the nuclei, and the numbers of TUNEL-positive cells were recorded [34]. MTT was used to analyze the cellular viability. Cells (1x10^6^ cells/well) were cultured on a 96-well plate at 37°C with 5% CO_2_. Then, 40 μl of MTT solution (2 mg/ml; Sigma-Aldrich) was added to the medium for 4 h at 37°C with 5% CO_2_. Subsequently, the cell medium was discarded, and 80 μl of DMSO was added to the wells for 1 h at 37°C with 5% CO_2_ in the dark. The OD of each well was observed at A490 nm via a spectrophotometer (Epoch 2; BioTek Instruments, Inc., Winooski, VT, USA) [35].

### 2.5 EdU incorporation assay

EdU staining was conducted using the BeyoClick™ EdU Cell Proliferation Kit with Alexa Fluor 594 (Beyotime, Cat. No: C00788L). Cells were washed with PBS. Fresh DMEM was added, and then, 10 μM EdU was added into the medium. The cells were incubated for 2 hours at 37°C/5% CO_2_. After the incubation, the cells were washed with PBS to remove the DMEM and the free EdU probe [36]. The cells were then fixed in 4% paraformaldehyde at room temperature for 30 minutes before being stained with DAPI for 3 minutes. After an additional wash in PBS, the cells were observed under an inverted microscope [37].

### 2.6 Mitochondrial ROS analysis

Flow cytometry was applied as a quantitative method for evaluating mitochondrial ROS levels according to a previous study. Cells were seeded onto 6-well plates [38]. Subsequently, the cells were isolated using 0.25% trypsin and then incubated with MitoSOX red mitochondrial superoxide indicator (Molecular Probes, USA) for 30 min in the dark at 37°C. Subsequently, PBS was used to wash cell two times, and then the cells were analyzed with a FACS Calibur Flow cytometer. Data were analyzed by FACS Diva software. The experiment was repeated three times to improve the accuracy [39].

### 2.7 Mitochondrial membrane potential (MMP) evaluation

To observe the mitochondrial potential, JC-1 staining (Thermo Fisher Scientific Inc., Waltham, MA, USA; Catalog No. M34152) was used [40]. Then, 10 mg/ml JC-1 was added to the medium for 10 minutes at 37°C in the dark to label the mitochondria. Normal mitochondrial potential showed red fluorescence, and damaged mitochondrial potential showed green fluorescence. The mPTP opening was measured via tetramethylrhodamine ethyl ester fluorescence according to a recent study [41].

### 2.8 ELISA

To analyze changes in caspase-9, caspase-9 activity kits (Beyotime Institute of Biotechnology, China; Catalog No. C1158) were used according to the manufacturer’s protocol. In brief, to measure caspase-9 activity, 5 μl of LEHD-p-NA substrate (4 mM, 200 μM final concentration) was added to the samples for 1 hour at 37°C [42]. Then, the absorbance at 400 nm was recorded via a microplate reader to reflect the caspase-3 and caspase-9 activities. To analyze caspase-3 activity, 5 μL of DEVD-p-NA substrate (4 mM, 200 μM final concentration) was added to the samples for 2 hours at 37°C. The levels of antioxidant factors, including GPX, SOD, and GSH, were measured with ELISA kits purchased from the Beyotime Institute of Biotechnology [43]. The experiments were performed in triplicate and repeated three times with similar results.

### 2.9 Nurr1 adenovirus transfection

Adenovirus-Nurr1 was transfected into cells to perform the gain-of-function assay. The pCMV6-Kan/Neo Nurr1 plasmids were obtained from OriGene Technologies, Inc. Then, 3.0 μg of the above plasmids were transfected into 293 T cells (2×10^4^ cells/well, National Infrastructure of Cell Line Resource) in DMEM with 10% FBS using Lipofectamine® 2000 (Invitrogen) [44]. After 48 hours, the supernatant was collected to obtain the Nurr1 adenovirus (Ad-Nurr1), which was transfected into A549 cells in Opti-MEM media supplemented with Lipofectamine® 2000 according to the manufacturer’s protocol. Transfection was carried out for 48 hours under 5% CO_2_ at 37°C. Then, Western blotting was performed to verify the transfection efficiency. Null adenovirus (Ad-ctrl) transfection was used as the negative control group [45].

### 2.10 Statistical analysis

Data are presented as the means ± S.E.M., and differences were deemed significant when P < 0.05. Data were analyzed using student t test for comparison between two groups, one-way ANOVA for comparison among three or more groups, and non-parameter comparative methods if needed.

## 3 Results

### 3.1 Overexpression of Nurr1 induces A549 lung cancer cell apoptosis and proliferation arrest

**Figure 1:**
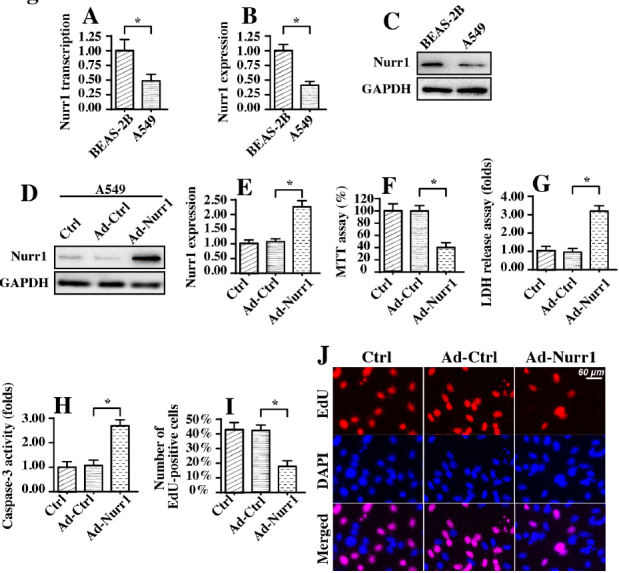
Nurr1 is downregulated in A549 lung cancer cells, and overexpression of Nurr1 promotes A549 cancer cell apoptosis. A. qPCR was used to analyze the transcription of Nurr1 in A549 lung cancer cells and BEAS-2B normal pulmonary epithelial cells. B-C. Proteins were isolated from cells, and the expression of Nurr1 in A549 cells and BEAS-2B cells was determined via western blotting. D-E. Nurr1-loaded adenovirus (Ad-Nurr1) and control adenovirus (Ad-Ctrl) were transfected into A549 cells. Then, western blotting was conducted to confirm the overexpression of Nurr1 mediated by Ad-Nurr1 in A549 cells. F. An MTT assay was used to observe alterations in A549 cell viability in response to Nurr1 overexpression. Nurr1-loaded adenovirus (Ad-Nurr1) and control adenovirus (Ad-Ctrl) were transfected into A549 cells. G. Results of an LDH release assay for A549 cells transfected with Ad-Nurr1 or Ad-Ctrl. H. Caspase-3 activity was measured in A549 cells. Nurr1-loaded adenovirus (Ad-Nurr1) and control adenovirus (Ad-Ctrl) were transfected into A549 cells. I-L. EdU staining was used to observe the influence of Nurr1 overexpression on cell proliferation. The number of EdU-positive cells was recorded. *p＜0.05.

To understand the regulatory role of Nurr1 in A549 lung cancer, qPCR and western blotting were used to determine the expression of Nurr1. As shown in Fig. 1A, compared to that in BEAS-2B normal pulmonary epithelial cells, the transcription of Nurr1 was downregulated in A549 cancer cells. In addition, Nurr1 expression was also reduced in A549 cancer cells compared to BEAS-2B normal pulmonary epithelial cells (Fig. 1B-C). These data suggest that Nurr1 is downregulated when normal epithelial cells differentiate into cancer cells. To explore the functional role of Nurr1 in the phenotypes of A549 lung cancer cells, Nurr1-loaded adenovirus (Ad-Nurr1) and control adenovirus (Ad-Ctrl) were transfected into A549 lung cancer cells. The overexpression efficiency was confirmed via western blotting, as shown in Fig. 1D-E. Subsequently, the viability of A549 cells transfected with Ad-Nurr1 was monitored. Compared to transfection with Ad-Ctrl, Ad-Nurr1 transfection reduced cell viability, as assessed via MTT assay (Fig. 1F). Similar results were also obtained in an LDH release assay. As shown in Fig. 1G, compared to Ad-Ctrl transfection, Ad-Nurr1 transfection evoked more LDH release into the medium, indicating that Nurr1 overexpression may promote cell death. This finding was further supported in an analysis of the activity of caspase-3. Compared to that in the Ad-Ctrl group, the activity of caspase-3 was significantly increased in A549 cells transfected with Ad-Nurr1 (Fig. 1H), reconfirming that Nurr1 overexpression triggered A549 death via apoptosis. In addition to cell death, proliferation was also evaluated using EdU staining. As shown in Fig. 1I-J, compared to the Ad-Ctrl group, the Ad-Nurr1 group had a lower percentage of EdU-positive cells, suggesting that Nurr1 overexpression impaired A549 cell proliferation.

### 3.2 Nurr1 overexpression triggers mitochondrial bioenergetic dysfunction

**Figure 2:**
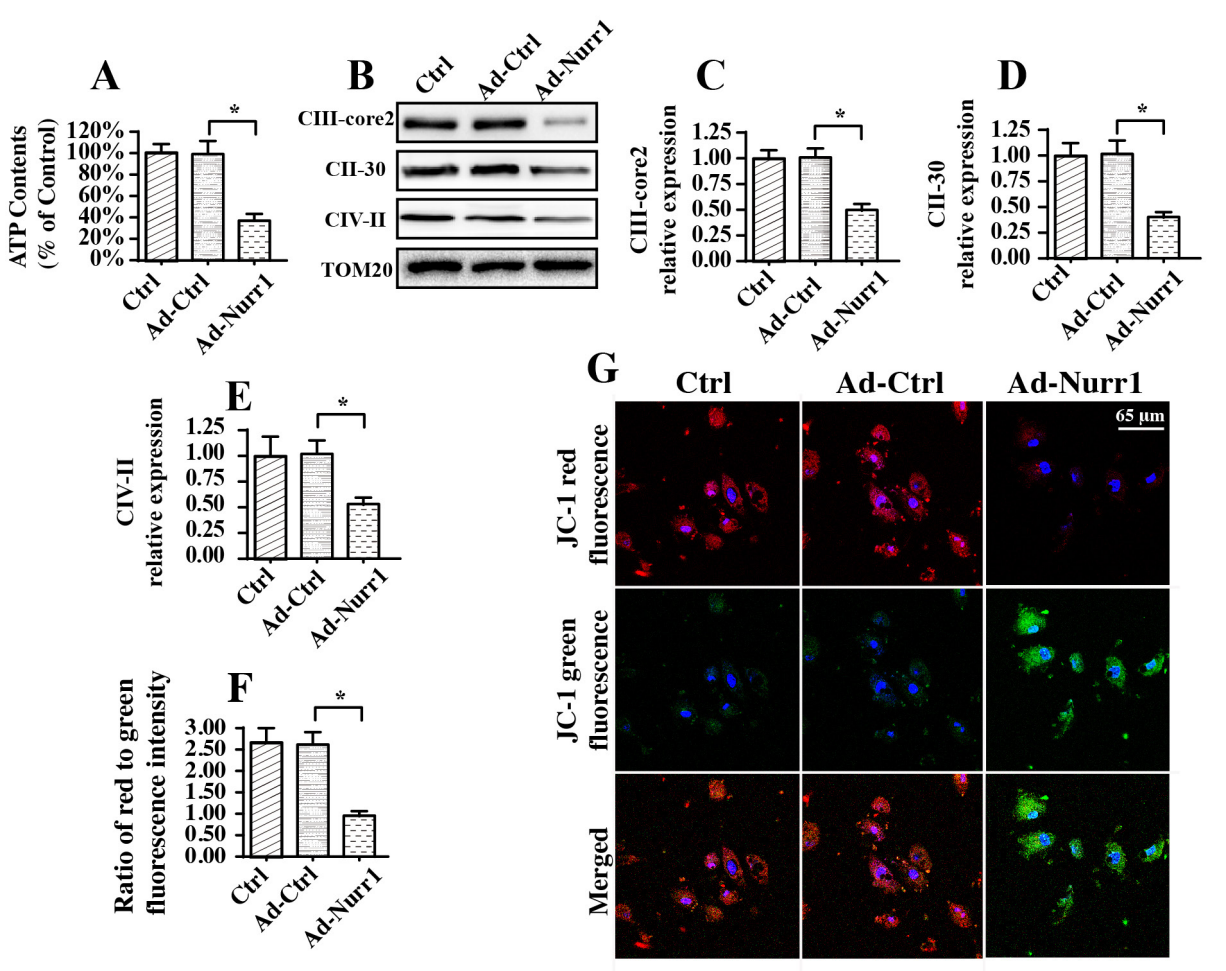
Nurr1 modulated mitochondrial bioenergetic dysfunction. A. ATP production was determined in A549 cells. Nurr1-loaded adenovirus (Ad-Nurr1) and control adenovirus (Ad-Ctrl) were transfected into A549 cells. B-E. Western blotting was used to observe alterations in the mitochondrial respiratory complex. Nurr1-loaded adenovirus (Ad-Nurr1) and control adenovirus (Ad-Ctrl) were transfected into A549 cells. F-G. A JC-1 probe was used to stain mitochondria. Then, the red fluorescence and green fluorescence of mitochondria were observed. Nurr1-loaded adenovirus (Ad-Nurr1) and control adenovirus (Ad-Ctrl) were transfected into A549 cells. *p＜0.05.

Next, experiments were performed to observe alterations in mitochondrial bioenergetics in A549 cells transfected with Ad-Nurr1. First, cell ATP production was measured to reflect mitochondrial energy production. Compared to Ad-Ctrl transfection, Ad-Nurr1 transfection reduced ATP production in A549 cells (Fig. 2A). Notably, mitochondria have the ability to generate ATP with the help of the mitochondrial respiratory complex [46, 47]. Accordingly, western blotting was used to observe changes in the expression of the mitochondrial respiratory complex. Compared to Ad-Ctrl transfection, Ad-Nurr1 transfection drastically reduced the levels of the mitochondrial respiratory complex (Fig. 2B-E), indicating that Nurr1 overexpression triggered mitochondrial respiratory dysfunction. In addition, cell energy metabolism is highly dependent on mitochondrial membrane potential [30]. Accordingly, a JC-1 probe was used to detect alterations in mitochondrial membrane potential. As shown in Fig. 2F-G, normal A549 cells exhibited more JC-1 red fluorescence than green fluorescence, indicative of high mitochondrial membrane potential. Interestingly, transfection of Ad-Nurr1 significantly reduced mitochondrial membrane potential, as evidenced by decreased red fluorescence and increased green fluorescence.

### 3.3 Nurr1 overexpression activates mitochondrial apoptosis

**Figure 3:**
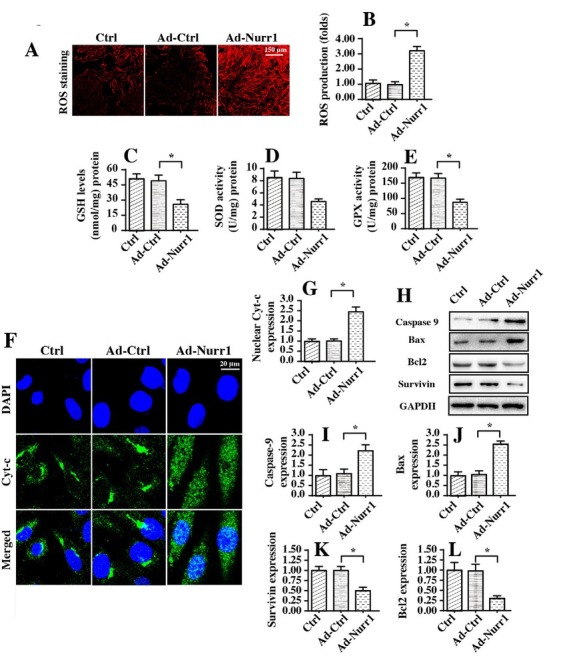
Nurr1 overexpression triggers mitochondrial apoptosis in A549 lung cancer cells. A-B. Cellular oxidative stress was determined using an immunofluorescence assay with a ROS probe. The fluorescence intensity of ROS was measured. C-E. ELISA was used to analyze the concentrations of cellular antioxidants, such as GSH, GOD and GPX. Nurr1-loaded adenovirus (Ad-Nurr1) and control adenovirus (Ad-Ctrl) were transfected into A549 cells. F-G. Results of an immunofluorescence assay for cyt-c liberation. DAPI was used to stain the nuclei. H-L. Proteins were isolated from A549 cells treated with Ad-Nurr1. Then, western blotting was used to evaluate the expression of mitochondrial apoptosis-related proteins such as caspase-9, Bax, Bcl2 and survivin. *p＜0.05.

Subsequently, we explored whether Nurr1 overexpression activated mitochondrial apoptosis in A549 cells. Mitochondrial apoptosis is characterized by mitochondrial oxidative stress, mitochondrial cyt-c liberation into the nucleus, and caspase-9 activation [46, 48]. First, an immunofluorescence assay was used to observe mitochondrial ROS production. Compared to Ad-Ctrl transfection, Ad-Nurr1 transfection significantly increased mitochondrial ROS production in A549 cells (Fig. 3A-B). To further analyze mitochondrial ROS overloading, we measured alterations in cellular antioxidants. As shown in Fig. 3 C-E, compared to Ad-Ctrl transfection, Ad-Nurr1 transfection reduced the concentrations of cellular antioxidants, including GSH, GOD and GPX. This information illustrated that Nurr1 overexpression triggered mitochondrial oxidative injury in A549 cells.

In addition to mitochondrial oxidative injury, we further observed alterations in cyt-c translocation into the nucleus (Fig. 3F-G). Through immunofluorescence studies, we found that cyt-c appeared in the cytoplasm in normal A549 cells. Interestingly, Ad-Nurr1 transfection promoted cyt-c liberation into the nucleus, indicating that Nurr1 overexpression evoked cyt-c migration. Subsequently, western blotting was used to confirm the alteration of mitochondrial apoptotic proteins. As shown in Fig. 3H-L, compared to Ad-Ctrl transfection, Ad-Nurr1 transfection upregulated the expression of caspase-9 and Bax. However, the expression of Bcl-2 and survivin were downregulated in response to Nurr1 overexpression. This information indicated that Nurr1 activated mitochondrial apoptosis in A549 cells.

### 3.4 Nurr1 overexpression promotes mitochondrial fission and inhibits mitochondrial fusion

**Figure 4:**
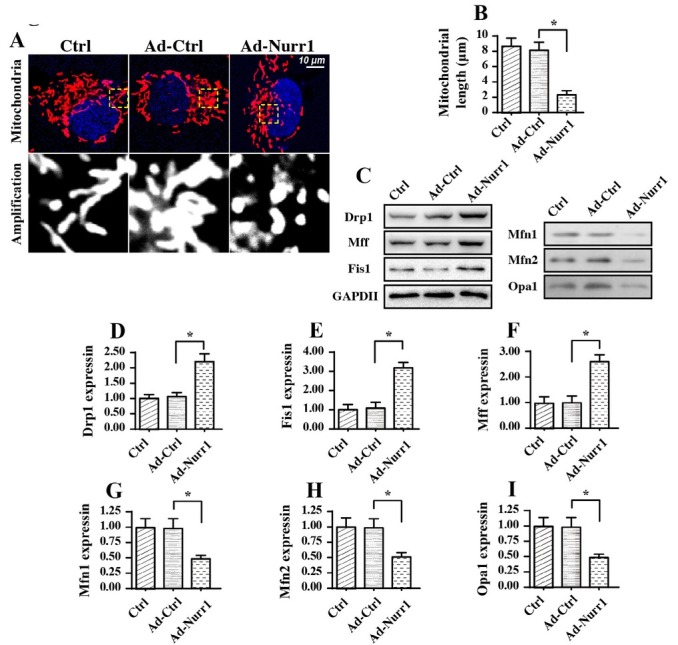
Nurr1 modulates mitochondrial fission and mitochondrial fusion in A549 cancer cells. A-B. Immunofluorescence for mitochondria and the mitochondrial structure was observed. Changes in average length of mitochondria were measured in response to Nurr1 overexpression. C-I. Proteins were isolated from A549 cells transfected with Ad-Nurr1, and the expression of mitochondrial fission factors, such as Drp1, Mff and Fis1, was measured. In addition, the levels of mitochondrial fusion factors, including Mfn1, Mfn2 and Opa1, were determined via western blotting in A549 cells transfected with Ad-Nurr1. *p＜0.05.

In addition to mitochondrial dysfunction, we also observed alterations in mitochondrial structure. First, an immunofluorescence assay was used to observe mitochondrial morphology. As shown in Fig. 4A-B, the mitochondria were interconnected in normal A549 cells, and the average mitochondria was ~8.2 μm in length. Interestingly, Ad-Nurr1 transfection induced the formation of mitochondrial debris, and the length of mitochondria was reduced to ~2.9 μm. This information indicated that Nurr1 overexpression disturbed mitochondrial structural homeostasis. Previous studies have reported that mitochondrial structure is closely regulated by mitochondrial fission and mitochondrial fusion. Therefore, western blotting analysis was conducted to evaluate alterations in parameters related to mitochondrial fission and fusion [47, 49]. As shown in Fig. 4C-I, compared to control Nurr1 expression, Nurr1 overexpression upregulated the levels of Drp1, Mff and Fis1, indicating activation of mitochondrial fission in response to Nurr1 overexpression. In contrast, the expression of Mfn1, Mfn2 and Opa1 was downregulated in Nurr1-overexpressing cells, indicating inhibition of mitochondrial fusion in response to Nurr1 overexpression. Altogether, these data indicated that Nurr1 overexpression induced an imbalance between mitochondrial fission and fusion.

### 3.5 Nurr1 activated the p53-Drp1 signaling pathway

**Figure 5:**
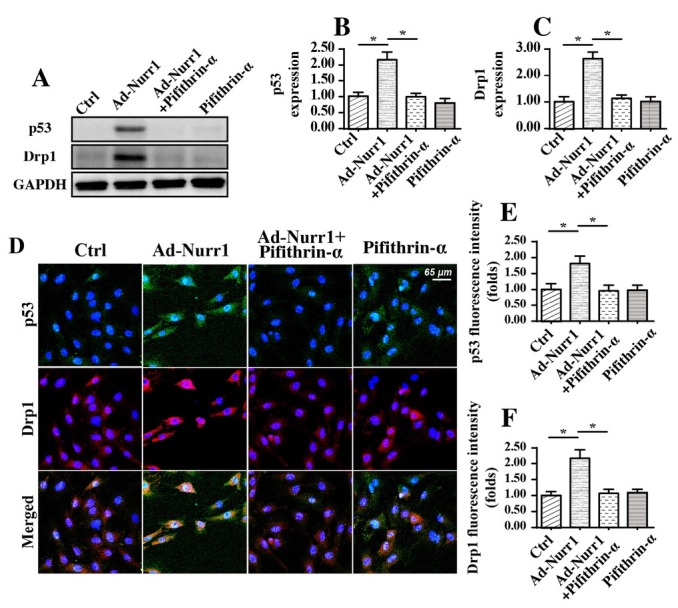
Nurr1 overexpression activates the p53-Drp1 signaling pathway. A-C. Western blotting was used to observe the influence of Nurr1 on p53 and Drp1. Nurr1-loaded adenovirus (Ad-Nurr1) and control adenovirus (Ad-Ctrl) were transfected into A549 cells. Pifithrin-α (PFTα), a blocker of p53, was incubated with Nurr1-overexpressing A549 cells. D-F. Results of an immunofluorescence assay for p53 and Drp1. Nurr1-loaded adenovirus (Ad-Nurr1) and control adenovirus (Ad-Ctrl) were transfected into A549 cells. Pifithrin-α (PFTα), a blocker of p53, was incubated with Nurr1-overexpressing A549 cells. *p＜0.05.

Considering the regulatory effects of Nurr1 on mitochondrial homeostasis, we wanted to determine the mechanisms by which Nurr1 modulated mitochondrial homeostasis in A549 cells. Previous studies have identified Drp1 as a master regulator of mitochondrial function, and increased Drp1 expression is associated with cancer cell death and invasion inhibition. In addition, p53 has been found to be the upstream mediator of mitochondrial homeostasis in lung cancer. Based on these data, we wanted to determine whether Nurr1 modulated mitochondrial homeostasis via the p53-Drp1 signaling pathway. First, western blotting was used to observe the influence of Nurr1 on p53 and Drp1 activation. As shown in Fig. 5A-C, compared to Ad-Ctrl transfection, Ad-Nurr1 transfection upregulated the expression of p53; this upregulation was accompanied by an increase in the expression of Drp1. This information illustrated that Nurr1 overexpression could activate the p53-Drp1 signaling pathway. Subsequently, the pathway blocker pifithrin-α (PFTα) was used in Nurr1-overexpressing cells to prevent p53 activation and Drp1 upregulation. The inhibitory effect of PFTα was confirmed via western blotting, as shown in Fig. 5A-C. This result was further supported via immunofluorescence. As shown in Fig. 5D-F, there was little expression of p53 and Drp1 in normal A549 cells. Interestingly, Nurr1 overexpression significantly increased the fluorescence intensity of p53 and Drp1 in A549 cells. In addition, PFTα treatment abolished the promotive effects of Nurr1 on Drp1 activation and p53 upregulation. Altogether, these data indicated that Nurr1 overexpression activated the p53-Drp1 signaling pathway in A549 cells.

### 3.6 The p53-Drp1 signaling pathway is involved in mitochondrial stress and cancer cell apoptosis

**Figure 6:**
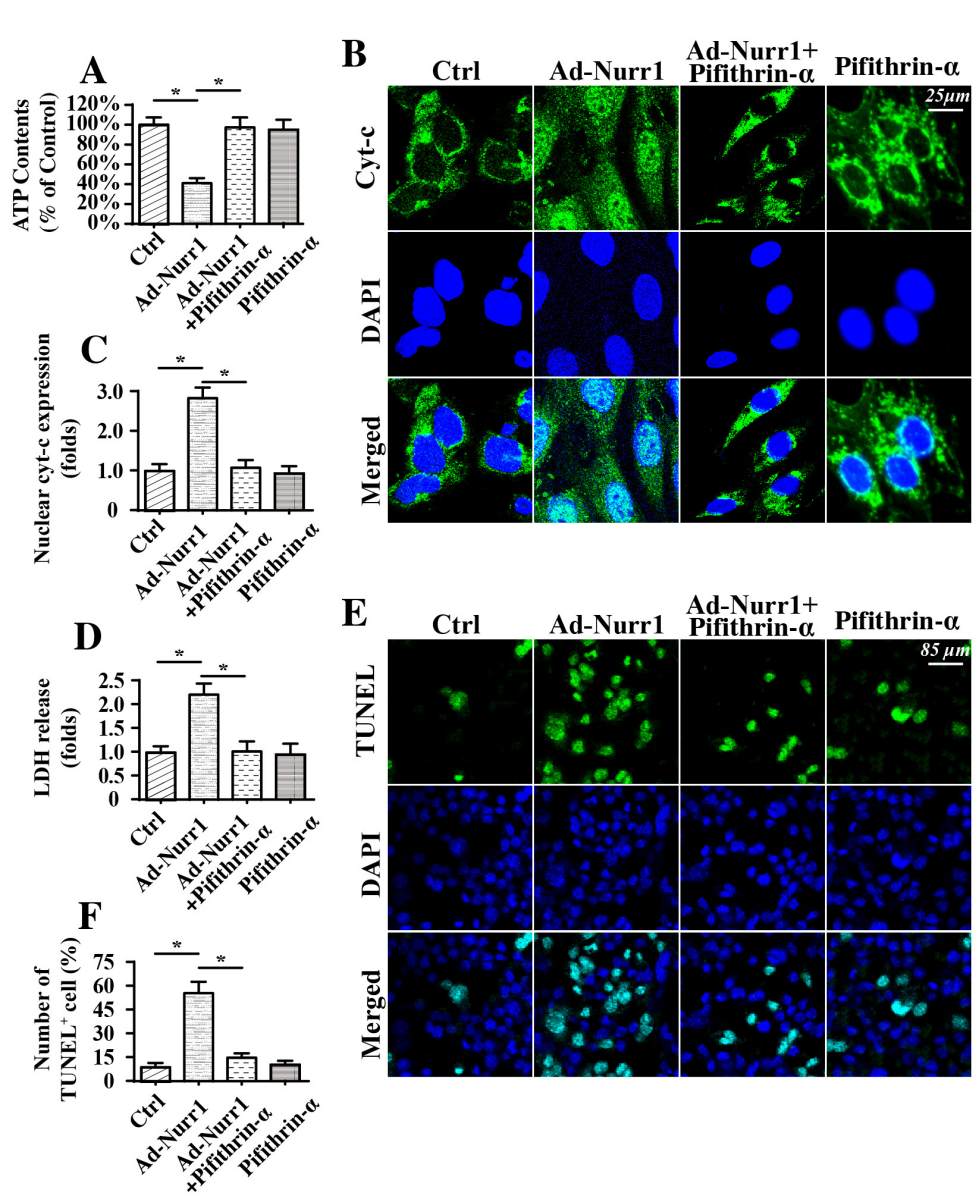
The p53-Drp1 axis is involved in Nurr1-mediated mitochondrial stress and cell death. A. ATP production was determined in response to Nurr1 overexpression. Pifithrin-α (PFTα), a blocker of p53, was used to prevent the activation of p53 and Drp1 in Nurr1-overexpressing cells. B-C. Results of an immunofluorescence assay for cyt-c in A549 cells. DAPI was used to stain the nuclei. D. Results of an LDH release assay for cell death in A549 cells treated with pifithrin-α (PFTα). E-F. TUNEL staining for apoptotic cells. The number of apoptotic cells was recorded. Nurr1-loaded adenovirus (Ad-Nurr1) and control adenovirus (Ad-Ctrl) were transfected into A549 cells. Pifithrin-α (PFTα), a blocker of p53, was incubated with Nurr1-overexpressing A549 cells. *p＜0.05.

Although we have confirmed the promotive effects of Nurr1 on the p53-Drp1 axis, it is unknown whether the p53-Drp1 signaling pathway is responsible for Nurr1-mediated mitochondrial stress and cancer cell apoptosis. Accordingly, we observed mitochondrial function and cell viability in A549 cells treated with PFTα. As shown in Fig. 6A, compared to control Nurr1 expression, Nurr1 overexpression reduced ATP production, and this effect could be reversed by PFTα, suggesting that Nurr1 reduced ATP production in a manner dependent on Nurr1 activity. In addition, mitochondrial apoptosis, as assessed via immunofluorescence analysis of cyt-c, also demonstrated that Nurr1 promoted cyt-c liberation into the nucleus (Fig. 6B-C). However, PFTα treatment repressed Nurr1-mediated cyt-c translocation. Moreover, Nurr1-mediated LDH release could also be abolished by PFTα treatment (Fig. 6D). This information indicated that Nurr1 induced mitochondrial stress in a manner dependent on the p53-Drp1 signaling pathway. To analyze A549 cell apoptosis, an TUNEL assay was performed. The number of apoptotic cells, as assessed via TUNEL assay, also increased in response to Nurr1 overexpression (Fig. 6E-F). Interestingly, PFTα treatment attenuated Nurr1-mediated A549 cell apoptosis. Taken together, our results indicated that the p53-Drp1 signaling pathway was required for Nurr1-mediated mitochondrial dysfunction and cell death in A549 lung cancer cells.

## 4 Discussion

In the present study, we provide ample data to support an important role of Nurr1 in the viability of A549 lung cancer cells. Our results showed that Nurr1 expression was significantly downregulated in A549 lung cancer cells compared to normal pulmonary epithelial cells. Interestingly, overexpression of Nurr1 via transfection of Nurr1-loaded adenovirus drastically increased the death index in A549 lung cancer cells. These results indicate that Nurr1 is a key antitumor protein that triggers cell death once normal cells convert into cancer cells. This finding is similar to that of a previous study. In patients with prostate cancer, Nurr1 gene transcription is significantly downregulated. Activation of Nurr1 delays the progression of skin cancer, gastric cancer, and leukemia. Notably, it is unknown whether overexpression of Nurr1 in normal lung tissue affects the viability and survival of normal cells. Additional studies are needed to verify whether excessive Nurr1 is harmful to normal lung tissue.

At the molecular level, Nurr1 has been reported to regulate regulatory T cell development through activation of the Foxp3 signaling pathway, and Nurr1-modified T cells have therapeutic potential for treating cancer [50]. In addition, Nurr1 modulates the inflammatory response in a manner dependent on the NF-κB pathway [51]. In addition, Nurr1 also impairs cancer prosurvival pathways such as the PI3K-Akt axis [52]. Other pathways, such as p53, HIF1 and VEGF-mediated angiogenesis pathways, are also regulated by Nurr1 [53]. In the present study, we found that Nurr1 overexpression modulated the expression of p53 and Drp1, which is in accordance with the results of previous studies. This information indicates that several signaling pathways related to cancer proliferation, survival and invasion are closely regulated by Nurr1. The reciprocal actions of Nurr1 on tumor signaling pathways highlight that the use of Nurr1 agonists may be a therapeutic strategy for cancer treatment.

The key finding of our study is that we revealed, for the first time, that Nurr1 acts as a tumor suppressor in lung cancer by modulating mitochondrial homeostasis. We found that Nurr1 overexpression induced mitochondrial damage, as evidenced by mitochondrial bioenergetics dysfunction, mitochondrial fission activation, mitochondrial fusion repression and mitochondrial apoptosis initiation. In agreement with our findings, previous studies have also reported links between mitochondrial dysfunction and Nurr1. Nurr1 modulates intracellular calcium balance and mitochondrial ATP production in stem cells[54]. In addition, Nurr1 is also involved in dopaminergic neurodegeneration through modulation of mitochondrial cyt-c translocation. In a model of Parkinson’s disease (PD), Nurr1 agonists affect mitochondrial membrane potential stabilization and intracellular ROS production in a manner dependent on PPARγ. In the present study, we further investigated the regulatory effects of Nurr1 on mitochondrial structure. Increased Nurr1 expression promoted mitochondrial fission and inhibited mitochondrial fusion, indicating disruption of mitochondrial dynamics, in A549 lung cancer cells. Similar to our findings, a recent study on a mouse model of cerebral ischemia/reperfusion also reported that Nurr1 upregulation was involved in the pathogenesis of cerebral reperfusion injury through activation of INF2-mediated mitochondrial fission [55]. Notably, ample evidence has indicated that excessive mitochondrial fission and decreased mitochondrial fusion are apoptotic signals for tumors such as those in pancreatic cancer, colorectal cancer, liver cancer, breast cancer, ovarian cancer, oral cancer, and gastric cancer [56, 57]. This finding provides evidence to explain the proapoptotic effects of Nurr1 on mitochondrial apoptosis and A549 cell apoptosis. However, little attention has been paid to the role of Nurr1-related mitochondrial dysfunction in cancer cell death. Accordingly, more studies are needed to investigate whether Nurr1-related mitochondrial fusion participates in cancer cell mitochondrial apoptosis. Besides, in the normal lung tissue, there is no study to explore the detailed role played by Nurr1 in cell viability. Although previous studies have demonstrated that Nurr1 expression is increased in cadmium-induced lung damage via activating apoptosis [58], the actions of Nurr1 on mitochondrial homeostasis and lung tissue homeostasis are unclear. Accordingly, more studies are required to verify the influence of Nurr1 in normal lung tissue.

Nurr1 induces mitochondrial stress via the p53-Drp1 signaling pathway. Blockade of the p53-Drp1 axis could attenuate the impacts of Nurr1 on mitochondrial damage and cell death. The influence of Drp1 on lung cancer cell death has been reported by several careful researchers. Drp1 downregulation is associated with the progression of human lung cancer [59], and increased Drp1 suppresses A549 lung cancer cell proliferation. In addition, p53 has been described to defend against the oncogenesis of lung cancer [60] through multiple effects, including apoptosis activation, proliferation repression, cancer metabolism management and immunoregulation. In the present study, our results demonstrated that p53 and Drp1 are downstream effectors of Nurr1. Activation of the p53-Drp1 pathway was responsible for Nurr1-mediated mitochondrial stress and cancer cell death. Moreover, mitochondrial apoptosis and mitochondria-induced apoptosis are totally different. Mitochondrial apoptosis is a cellular death process that is accompanied with mitochondrial dysfunction. However, mitochondria-induced apoptosis is highly modulated by mitochondria and apoptosis could be reversed via sustaining mitochondrial homeostasis. In the present study, we observed the influence of Nurr1 on mitochondria-induced apoptosis. These data help us to establish a novel signaling pathway in the regulation of mitochondrial function and cancer cell viability. However, more clinical data are needed to support our conclusions.

Altogether, our results confirmed the antitumor effects of Nurr1 in A549 lung cancer cells. Overexpression of Nurr1 induced A549 lung cancer cell death by activating mitochondrial dysfunction, as evidenced by mitochondrial respiratory dysfunction, mitochondrial redox imbalance, mitochondrial membrane potential reduction and mitochondrial apoptosis activation. Mechanistically, Nurr1 modulated mitochondrial function and cell viability via the p53-Drp1 signaling pathway. This finding introduces a novel cancer suppressor for A549 lung cancer and paves the way for new treatment strategies for lung cancer.
